# Excitonic insulator powers room-temperature ultra-sensitive visible to terahertz detection

**DOI:** 10.1038/s41377-025-01828-8

**Published:** 2025-04-02

**Authors:** Yi Wu, Wenjie Deng, Yongzhe Zhang

**Affiliations:** 1 Key Laboratory of Optoelectronics Technology of Education, School of Information Science and Technology, Beijing University of Technology, 100124 Beijing, China; 2 College of Materials Science and Engineering, Beijing University of Technology, 100124 Beijing, China

**Keywords:** Optics and photonics, Electronics, photonics and device physics

## Abstract

Phase transitions induce significant changes in the electrical and photonic properties of materials. Ultra-sensitive photodetectors leveraging material phase transitions can be realized near the transition temperature. Photodetectors based on Ta_2_NiSe_5_, a room-temperature excitonic insulator phase transition material, exhibit exceptional performance from visible to terahertz frequencies. Specifically, in the terahertz range, the electronic bandwidth is 360 kHz, and the specific detectivity (D*) reaches 5.3 × 10^11^ cm·Hz^1/2^·W^−1^. The van der Waals heterostructure of Ta_2_NiSe_5_/WS_2_ further enhances performance.

Photodetectors are pivotal in transducing optical signals into electronic data, thereby enabling a wide array of technologies and applications across various domains, such as communication, imaging, sensing, and measurement^1,2^. Silicon-based photodetectors, including CMOS image sensors and charge-coupled device (CCD) sensors, have achieved significant commercial success. The CCD technology was recognized with the Nobel Prize in 2009. However, their performance is limited to the visible and near-infrared wavelength due to the intrinsic bandgap limitations of silicon and its photoresponse mechanism. Infrared (IR) and Terahertz (THz) photodetectors are essential for technological advancements in security, healthcare, environmental monitoring, and industrial applications, offering unique capabilities beyond other wavelengths^3,4^. Consequently, the study of IR and THz photodetectors has emerged as a prominent area of research. Photodetectors are commonly based on photonic and thermal mechanisms. Achieving IR and THz detection with photonic photodetectors necessitates the use of narrow bandgap materials; however, these materials typically exhibit high dark current at room temperature, requiring cooling, which is economically unfeasible^5,6^. Conversely, thermal-effect photodetectors, while operable at room temperature, generally display low sensitivity, relatively slow response speeds, and limited electrical bandwidth due to inherent operating mechanism^7,8^. Another approach leverages the changes in electrical and optical properties induced by phase transitions in materials, which can be exploited in photodetection. Since phase transitions induce abrupt changes in material properties, they can be harnessed for high sensitivity applications. However, most materials lack phase transition temperatures within the room temperature range^9,10^.

In a recent publication in Light: Science & Applications, the research team led by Prof. Z. Huang at the Shanghai Institute of Technical Physics, Chinese Academy of Sciences, has proposed and demonstrated an ultrasensitive Ta_2_NiSe_5_-based room-temperature phase transition photodetector^11^. This device exhibits exceptional sensitivity in the terahertz band, achieving a specific detectivity (D*) of up to 5.3 × 10^11 ^cm·Hz^1/2^·W^−1^ and an electrical bandwidth of 360 kHz, surpassing the state-of-the-art room-temperature terahertz detectors by one to two orders of magnitude.

The mechanism of this photodetector is the phase transition between semimetal and excitonic insulator (EI) at room temperature (326 K) and thus can be applied to highly sensitive photodetectors as shown in Fig. 1^12,13^. For the phase transition process of Ta_2_NiSe_5_, Huang et al. characterized it by Raman spectroscopy analysis at variable temperatures, and the additional Raman peaks appearing at different temperatures proved the occurrence of the phase transition. And Hall tests at different temperatures revealed that the carrier concentration and mobility of the material changed dramatically before and after the phase transition. Thus leading to the sensitive optical response of the material.

**Fig. 1:**
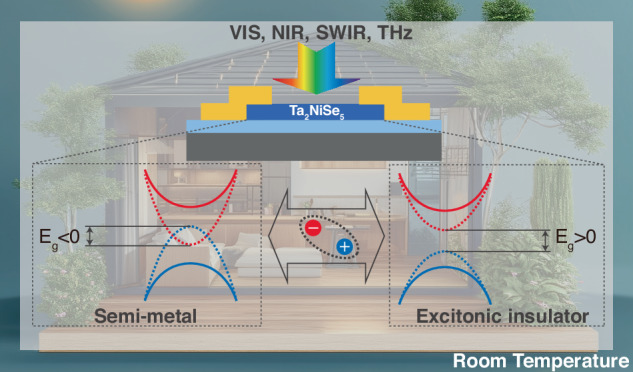
**Schematic of the Ta**_**2**_**NiSe**_**5**_ **photodetector**. Exciton formation leads to a transition from a semi-metal to an insulator that can significantly alter the electronic and optical properties

Huang et al. developed Ta_2_NiSe_5_-based detectors exhibiting ultra-high responsiveness to visible, near-infrared, short-wave infrared, and terahertz frequencies at room temperature. As the photoresponse is proportional to mobility and inversely proportional to carrier concentration, the rate of change for both peaks near the phase transition temperature, thereby achieving the maximum photocurrent. The electrical bandwidth in terahertz waveband (0.14 THz) was obtained as 360 kHz. Responsivity achieved peak at room temperature, with 4.7 × 10^3 ^A ∙ W^−1^ (0.344 THz). The rising and falling time are estimated to be 500 ns and 1.4 μs. Specific detectivity (D*) in the terahertz band up to 5.3 × 10^11 ^cm·Hz^1/2^·W^−1^ at 11,600 μm. This performance is already superior to the state of the art room temperature terahertz photodetectors.

Furthermore, Ta_2_NiSe_5_ is a two-dimensional material, offering the advantage of having no dangling bonds, allowing it to form heterojunctions with other materials without lattice mismatch concerns, thereby suppressing dark current and enhancing performance^14,15^. Consequently, Huang et al. formed a heterojunction of Ta_2_NiSe_5_ and WS_2_, which reduces dark current and noise through the generation of potential barriers at the interface. At 0.024 THz and 0.171 THz, the heterojunction device achieves maximum D* values of 7.0 × 10^11 ^cm·Hz^1/2^· W^−1^ and 3.9 × 10^11 ^cm·Hz^1/2^·W^−1^ at V_ds_ = 2.5 V, respectively. More importantly, the introduction of WS_2_ enables tunable device performance via gate voltage and enhances performance across the visible, near-infrared, and short-wave infrared spectra. Thus, the detector offers performance advantages over current photodetectors across a wide spectral range from visible to terahertz.

Overall, this article highlights Ta_2_NiSe_5_ as an ideal material for highly sensitive room-temperature photodetectors due to its phase transition temperature aligning with room conditions. Additionally, its two-dimensional nature offers extensive potential for future development. Photodetectors based on excitonic insulator phase transitions hold significant promise. This research is poised to yield more cost-effective, high-performance photodetectors.
